# The Effect of a New Glucose–Methotrexate Conjugate on Acute Lymphoblastic Leukemia and Non-Hodgkin’s Lymphoma Cell Lines

**DOI:** 10.3390/molecules26092547

**Published:** 2021-04-27

**Authors:** Marta Woźniak, Sebastian Makuch, Gabriela Pastuch-Gawołek, Jerzy Wiśniewski, Wiesław Szeja, Martyna Nowak, Monika Krawczyk, Siddarth Agrawal

**Affiliations:** 1 Department of Pathology, Wroclaw Medical University, 50-367 Wroclaw, Poland; marta.wozniak@umed.wroc.pl (M.W.); sebastian.mk21@gmail.com (S.M.); martyna.nowak96@o2.pl (M.N.); 2 Department of Organic Chemistry, Bioorganic Chemistry and Biotechnology, Faculty of Chemistry, Silesian University of Technology, 44-100 Gliwice, Poland; gabriela.pastuch@polsl.pl (G.P.-G.); wieslaw.szeja@adres.pl (W.S.); 3 Biotechnology Centre, Silesian University of Technology, 44-100 Gliwice, Poland; 4 Central Laboratory of Instrumental Analysis, Wroclaw University of Science and Technology, 50-370 Wroclaw, Poland; jerzy.wisniewski@pwr.edu.pl; 5 Department and Clinic of Internal Medicine, Occupational Diseases, Hypertension and Clinical Oncology, Wroclaw Medical University, 50-556 Wroclaw, Poland

**Keywords:** warburg effect, anticancer drugs, hematologic malignancies, drug design and discovery, glucose metabolism, glycoconjugates, methotrexate, targeted therapy

## Abstract

Patients with hematologic malignancies require intensive therapies, including high-dose chemotherapy. Antimetabolite–methotrexate (MTX) has been used for many years in the treatment of leukemia and in lymphoma patients. However, the lack of MTX specificity causes a significant risk of morbidity, mortality, and severe side effects that impairs the quality of patients’ life. Therefore, novel targeted therapies based on the malignant cells’ common traits have become an essential treatment strategy. Glucose transporters have been found to be overexpressed in neoplastic cells, including hematologic malignancies. In this study, we biologically evaluated a novel glucose–methotrexate conjugate (Glu–MTX) in comparison to a free MTX. The research aimed to assess the effectiveness of Glu–MTX on chosen human lymphoma and leukemia cell lines. Cell cytotoxicity was verified by MTT viability test and flow cytometry. Moreover, the cell cycle and cellular uptake of Glu–MTX were evaluated. Our study reveals that conjugation of methotrexate with glucose significantly increases drug uptake and results in similar cytotoxicity of the synthesized compound. Although the finding has been confined to in vitro studies, our observations shed light on a potential therapeutic approach that increases the selectivity of chemotherapeutics and can improve leukemia and lymphoma patients’ outcomes.

## 1. Introduction

Chemotherapeutic agents have remained the mainstay of hematologic malignancies therapy for decades, and in combination with radiotherapy (RT), these modalities continue to be the first-line option for most entities [1]. The treatment yields high cure rates in many diseases such as lymphoblastic leukemias or Hodgkin lymphomas; however, dose-dependent severe side effects resulting from lack of selectivity remain a significant drawback of the therapy [2]. The foremost reason for the cessation of conventional chemotherapy is not the lack of efficacy but the resulting toxicity. Well-recognized acute side effects include pancytopenia that leads to severe bleeding, incurable infections, and organ damage. Cardiovascular complications and second malignancies are among the most frequent and potentially life-limiting late effects. Other long-term adverse outcomes, particularly impaired organ function, infertility, fatigue, and pulmonary complications, are growing reasons for prolonged morbidity and mortality after effective first-line therapy in hematologic cancers.

The adverse outcomes limit the curative potential of conventional therapeutic modalities. Although young and fit cancer patients generally qualify for these treatments, the increasing number of elderly and frail individuals, as well as patients with multimorbidities, are commonly excluded from these therapeutic approaches. These facts emphasize the unmet need for potent yet safe treatments.

Methotrexate (MTX) is a cytostatic drug that is widely used to treat a wide variety of hematological cancers, either alone or in combination with other anticancer agents [3]. This compound competitively inhibits dihydrofolate reductase (DHFR), a crucial enzyme that produces tetrahydrofolate cofactors essential for DNA synthesis [4]. Despite its efficacy, this compound is characterized by low specificity and high toxicity to healthy cells.

In the last decades, notable advances in our understanding of disease-specific biologic and genetic features, courtesy of basic science research, have profoundly transformed the treatment landscape for hematologic malignancies with the introduction of targeted therapies. The accumulating evidence implies that we will observe a further shift from conventional chemo- and radiotherapy to preferred targeted therapies [2]. In an effort to improve the selectivity of methotrexate, MTX–AuNP (gold nanoparticles) and MTX–HA (hyaluronic acid) and their conjugates have been developed and studied [5,6]. The studies proved that the conjugation of MTX with a macromolecule or nanoparticles can be used as an invaluable tool in targeted cancer treatment.

To keep up with the fast multiplication and high-energy requirement, tumor cells significantly intensify their glucose dependence and metabolism [7,8]. Remarkably, malignant cells shift their metabolism from aerobic oxidation to anaerobic glycolysis, regardless of the oxygen concentrations. A direct implication of this metabolic transformation is lower energy production, resulting in an increase in facilitative glucose transporters (GLUT, gene family SLC2) on the cellular membrane [9]. Since GLUTs are overexpressed in numerous cancers, including hematologic malignancies [7,10], they provide a selective mechanism to target neoplastic cells. Noteworthy, targeted therapy is less toxic toward normal cells, compared to systemic therapy. Based on this information, one of the possible ways to reduce the toxicity of chemotherapy drugs is to use the differences between healthy and cancer cells to design prodrugs that will reduce drug toxicity before reaching the target cell, facilitate its transport into cancer cells through overexpressed proteins whose task is the transport of specific structural elements, and release the active form of the drug in the intracellular microenvironment.

**Figure 1:**
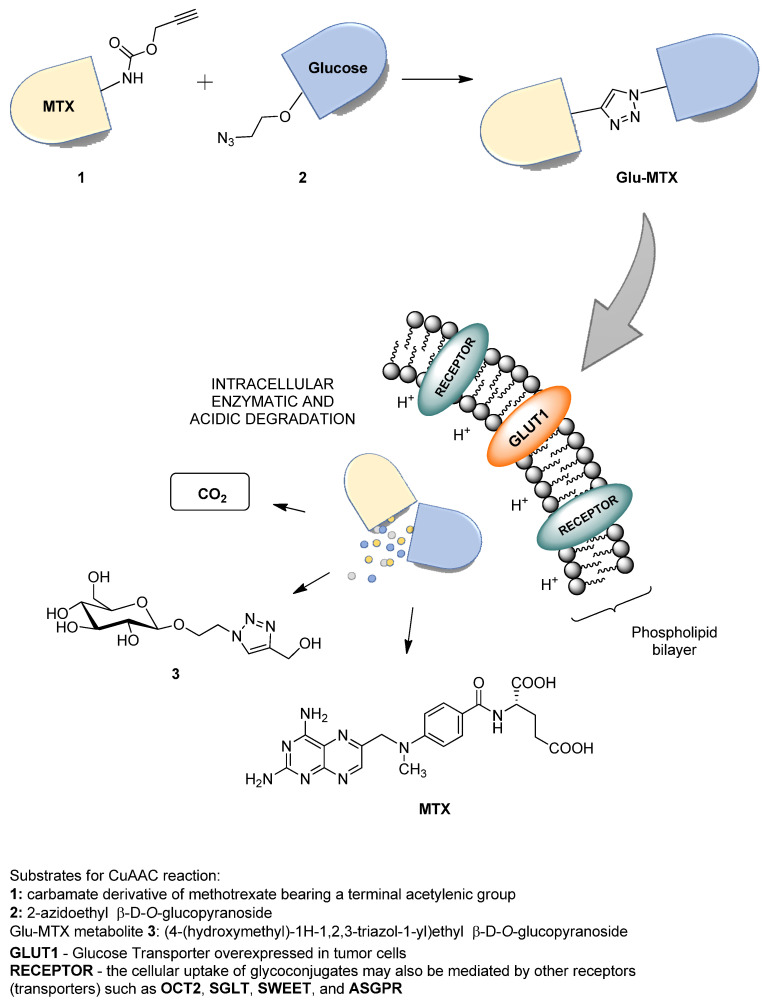
Scheme of Glu–MTX synthesis via copper-catalyzed 1,3-dipolar azide–alkyne cycloaddition (CuAAC), its transport into the cancer cell through the overexpressed GLUT1 transporters, and intracellular enzymatic and acidic degradation leading to the controlled release of biologically active metabolites (MTX and compound **3**).

The proposed solution focuses on improving the bioavailability and selectivity of methotrexate by attaching a sugar fragment to its structure. In the tested glucose–methotrexate conjugate (Glu–MTX), the sugar fragment facilitating the targeted uptake of the therapeutic agent is connected with the therapeutic agent such as methotrexate (MTX) via a linker characterized by lability under the action of intracellular hydrolytic enzymes. The mentioned linker is attached to the sugar unit via a glycosidic bond susceptible to degradation in the presence of intracellular glycosylhydrolases, and, contrastingly, it binds methotrexate via an easily hydrolyzed carbamate bond [8]. Such a designed structure of the tested compound allows for the assumption that after penetrating into the cell, Glu–MTX undergoes degradation with the release of the active form of the drug (MTX) and metabolites such as carbon dioxide and the 1,2,3-triazole derivative of glucose **3**, and the latter one may degrade further over time (Figure 1). An additional advantage of this conjugate is the fact that the glucose derivative **3**, containing the 1,2,3-triazole unit, released in the cell as a result of enzymatic hydrolysis, also has a slight cytotoxic activity [11]. Glycoconjugate Glu–MTX may be prepared in an efficient manner using one of the so-called click-chemistry reaction, the copper-catalyzed 1,3-dipolar cycloaddition of the azide to a terminal alkyne bond (CuAAC) in a variant developed by Sharpless and used for the synthesis of a wide range of biologically active compounds [12,13,14,15].

In the current work, we have examined a Glu–MTX conjugate targeting GLUT1 in hematologic malignancies, namely, acute lymphoblastic leukemia and non-Hodgkin’s lymphoma, and compared its effect with methotrexate, a chemotherapeutic agent widely applied in the treatment of leukemia and lymphoma.

## 2. Results

### 2.1. Glu–MTX Exerts a Similar Cytotoxic Effect on Lymphoma and Leukemia Cell Lines Compared to MTX

**Figure 2:**
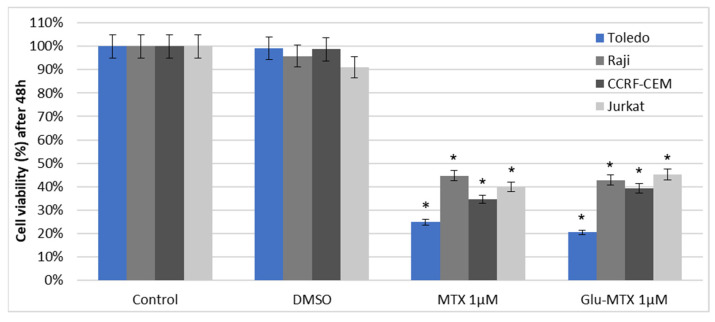
Cell viability after 48 h of incubation with 1 µM of MTX and Glu–MTX measured by MTT assay. DMSO was used as a vehicle. The statistical analysis of differences between control and treated sample was performed using an independent samples *t*-test. * *p*  <  0.05 was indicated as statistical significance, comparing the viability of control cells with MTX and Glu–MTX-treated cells. Data are expressed as the mean ± S.D of three separate experiments.

**Figure 3:**
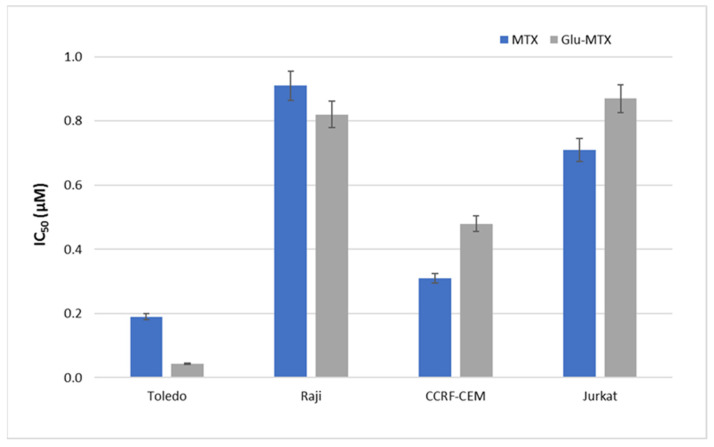
Comparison of IC_50_ activity of MTX and Glu–MTX. Incubation time 48 h.

To evaluate the cytotoxic effect of Glu–MTX and compare it with free MTX, the MTT assay was performed on four hematologic cancer cell lines. The results showed that the Glu–MTX compound is cytotoxic in vitro. The comparative results of a survival evaluation of cells (IC50) are shown (Figure 2 and Figure 3) for different hematological tumor cell lines treated with methotrexate or glucose–methotrexate derivative (Glu–MTX). The cytotoxic effect of the glucose–methotrexate conjugate is similar to that of unmodified methotrexate.

### 2.2. Glu–MTX Induces Early and Late Apoptosis in Lymphoma and Leukemia Cell Lines and Displays a Similar Apoptotic Effect to Free MTX

**Figure 4:**
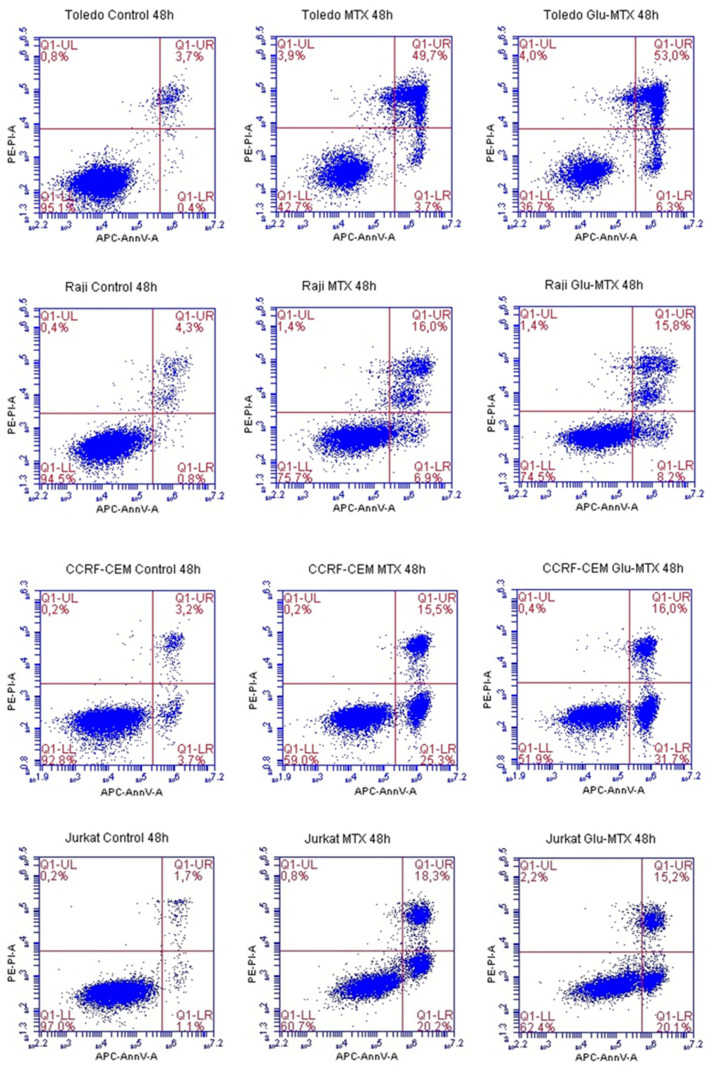
Representative images of flow cytometry analysis of alive (LL, lower left), early apoptotic (LR, lower right), late apoptotic (UR, upper right), and dead (UL, upper left) Toledo, Raji, CCRF–CEM, and Jurkat cells after incubation with 0.1 µM of MTX and Glu–MTX for 48 h.

**Figure 5:**
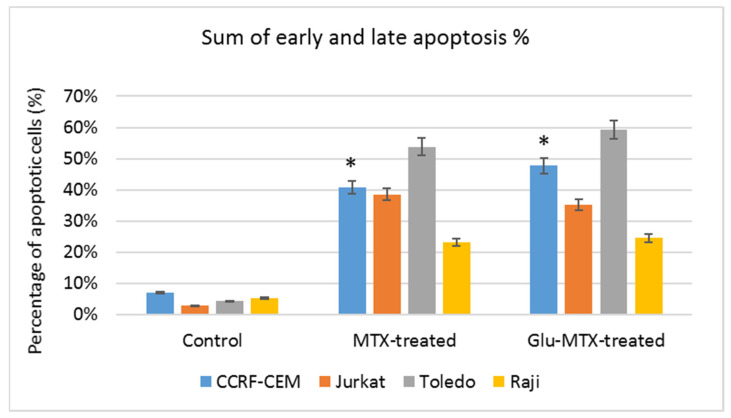
The percentage of early and late apoptotic cells after treatment with MTX and Glu–MTX. The statistical analysis of differences between control and treated sample was performed using an independent samples *t*-test. * *p*  <  0.05 was indicated as statistical significance, comparing MTX-treated cells with Glu–MTX-treated.

The apoptosis rate of Glu–MTX-treated cells was compared with MTX-treated cells and analyzed by flow cytometry. The sum of early and late apoptosis varied at 24.5% for Raji, 35.5% for Jurkat, 47.7% for CCRF–CEM, and 59.2% for Toledo cell line, after the treatment with Glu–MTX for 48 h (Figure 4 and Figure 5). Compared to free MTX (40.8%), the Glu–MTX induced apoptosis by 47.7% in the acute lymphoblastic leukemia CCRF–CEM cell line.

### 2.3. Glu–MTX Displays a Similar to MTX Mechanism of Action and Inhibits DNA Synthesis in the S Phase of the Cell Cycle

**Figure 6:**
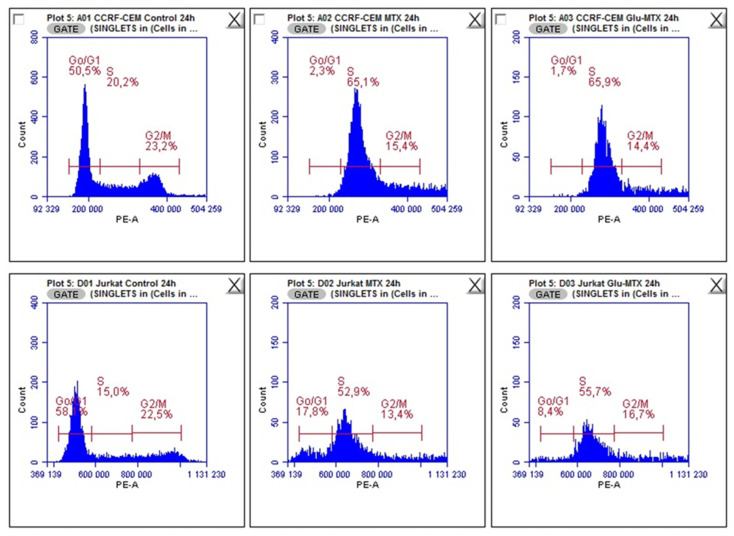
Cell cycle analysis of CCRF–CEM and Jurkat cells after treatment with 0.1 µM MTX and Glu–MTX for 24 h. For leukemia cells, cell cycle arrest in the S phase is characterized by the action of both the Glu–MTX conjugate and free methotrexate.

**Figure 7:**
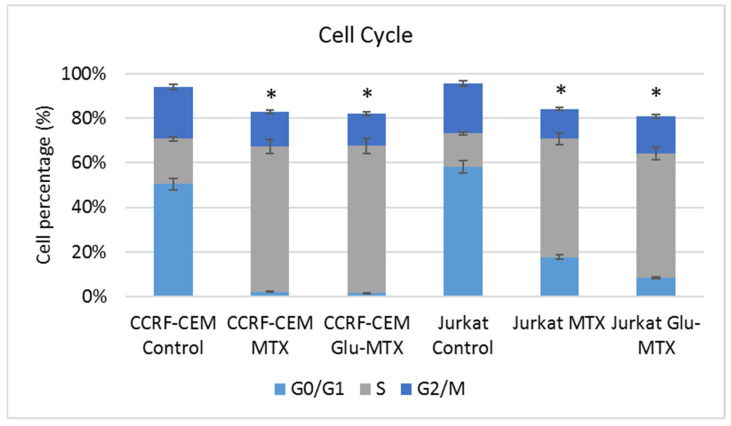
Comparative analysis between MTX and Glu–MTX-treated CCRF–CEM and Jurkat cells G0/G1, S, and G2/M phases. The statistical analysis of differences between control and treated samples was performed using ANOVA Kruskal–Wallis test. * *p*  <  0.05 was indicated as statistical significance, comparing MTX and Glu–MTX-treated cells with control.

To determine whether Glu–MTX displays the same mechanisms of action as MTX, its effect on cell cycle progression was examined on leukemia CCRF–CEM and Jurkat cell lines. The analysis showed that the 24 h treatment with Glu–MTX results in an S-phase arrested cell population (Figure 6 and Figure 7). This result indicates that both compounds affect the cell cycle by arresting the cells in the S phase and display a similar mechanism of action. The most plausible explanation of this phenomenon could be that the cleavage of Glu–MTX and the release of free MTX occurs in the intracellular compartment. The results from lymphoma cell lines (Raji and Toledo) showed no changes in the cell cycle after the treatment with MTX and Glu–MTX.

### 2.4. The Cellular Uptake of Glu–MTX Is Significantly Higher in CCRF–CEM Acute Lymphoblastic Leukemia Cells Compared to Free MTX

**Figure 8:**
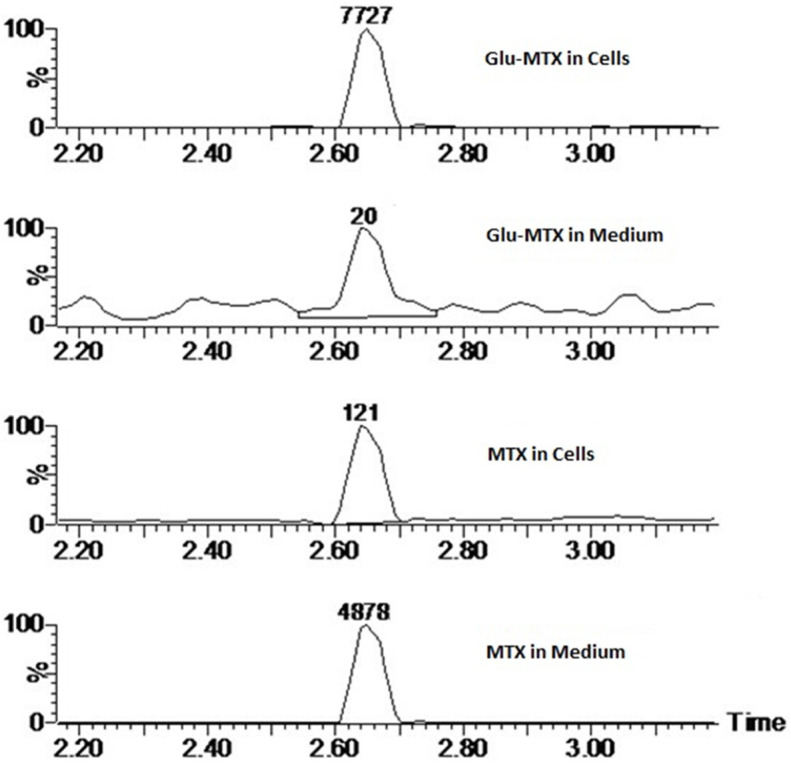
Extracted ion chromatograms of MTX (455.18 *m*/*z*) in cell extracts and culture medium. The peak area was shown above the peaks.

To investigate the cellular transport of Glu–MTX and answer the question of whether the conjugation of glucose improves the accumulation of the compound in the intracellular compartment, a mass spectrometry analysis was performed. The results showed that the Glu–MTX is approximately 63 times more preferentially accumulated in the intracellular compartment of CCRF–CEM cells (which were characterized by increased apoptotic rate), compared to MTX (Figure 8). This result supports the hypothesis that glucose conjugates exploit the Warburg’s effect and preferentially accumulate in cancer cells, contradistinctively to well-established nonconjugated agents that lack target specificity.

## 3. Discussion

The use of chemotherapy and autologous stem cell transplantation and the introduction of therapeutic agents, including proteasome, kinase, and histone deacetylase inhibitors, as well as immunomodulatory drugs, have enhanced clinical outcomes in patients with hematologic malignancies. However, a large portion of patients will discontinue the therapy due to adverse outcomes and/or eventually relapse, highlighting the urgent need for potent and safe therapeutic alternatives [16]. The bulk of these agents have significant toxicities that limit their dosing and potential for use in combination therapy. Although the introduction of targeted therapies has significantly enhanced outcomes for patients with hematologic malignancies, the unmet medical need remains. The burden of adverse effects resulting from systemic chemotherapy is heavy; hence, it would be desirable to develop a novel class of therapeutics that could combine conventional chemotherapeutic agents’ high efficacy and versatility with low systemic toxicity. Our goal was to build upon glucose derivatives’ success story by developing a novel carbohydrate conjugate that could exploit glucose transporters to target a range of hematologic malignancies selectively [17]. Here, we describe the generation and in vitro characterization of a glucose-methotrexate conjugate, a glucose transporter targeting agent with a unique combination of antitumor activities.

This study showed that the ability of Glu–MTX to induce apoptosis in vitro was slightly more prominent than MTX’s in acute lymphoblastic leukemia CCRF–CEM cell line. Moreover, the compound exhibited potent activity in low doses, comparable to free MTX. However, increased uptake of Glu–MTX apparently did not translate into a significantly higher cytotoxic and apoptotic effect, which serves as an indication that the conjugate is less cytotoxic, compared to free MTX, which in turn supports the results of our previous study [11]. Another possible explanation for this could be the fact that the drug uptake was measured after 6 h, while the apoptotic effect was evaluated after 48 h, which suggests that efflux of free MTX originated from Glu–MTX to the outside of the cells could have occurred. Additionally, Glu–MTX affected the cell cycle by arresting the cells in the S phase, which indicates that the release of free MTX disassembled from Glu–MTX occurs in the intracellular compartment. Moreover, the uptake of Glu–MTX in leukemia cells was 63 times more efficient, compared to free MTX after 6 h of incubation. This result proves conclusively that the conjugation of glucose to MTX improves its selectivity and transport in leukemia cancer cells.

Nevertheless, our study bears several limitations. Firstly, the effect of Glu–MTX was not evaluated on primary malignant cells and normal hematopoietic cells. Moreover, because hematological malignancies are a heterogeneous group of neoplastic disorders, a larger panel of cell lines would be more desirable. Furthermore, since MTX affects folic acid metabolism, it can affect erythrocytes or some other healthy cells that express folate receptors. It would be valuable to measure the uptake of Glu–MTX by different healthy cells in different time points, as well as MTX’s metabolites (e.g., folate–polyglutamate molecule levels) after the treatment. Yamauchi et al. [18] indicated that time-dependent change in MTX efflux in human leukemia cells corresponds with MTX activity. Moreover, according to the investigations of Walton et al. [19], circadian oscillations impact cancer cell metabolism and change the GLUT transporters’ expression. Therefore, the above-mentioned findings may be important factors affecting Glu–MTX mechanism.

Lastly, further investigation is needed to determine whether the GLUT1 expression correlates with the increased drug uptake in leukemia and lymphoma cell lines. Notwithstanding the significantly higher cellular uptake of Glu-MTX, we cannot state that the transport of the conjugate is mediated exclusively by GLUT transporters. Previous studies have highlighted a potential role of sodium-driven glucose symporters (SGLTs), organic cation transporter 2 (OCT2), and SWEET transporters in the cellular transport of glycoconjugates [20,21]. In future research, it would be eligible to include studies of cell sensitization with insulin prior to Glu–MTX incubation to check whether insulin can promote GLUT transporters overexpression in lymphoma and leukemia cells to increase the cellular uptake of the conjugate. These investigations are well-known in different cancers, such as breast cancer [22,23]. Drawing on the example of several successful conjugates of glucose utilized in hematologic cancer treatment [24,25,26], with their favorable pharmacokinetic and safety profile, a functional Glu–MTX conjugate could offer the prospect of highly successful therapy in GLUT-abundant hematologic malignancies that might also be used readily in combination with other modalities to improve clinical outcomes. On the basis of the reported findings, Glu–MTX has shown potent in vitro activity in acute lymphoblastic leukemia and non-Hodgkin’s lymphoma cell line models. Therefore, it presents an exciting opportunity for a novel class of compounds that could combine the high efficacy and versatility of conventional chemotherapeutic agents with low systemic toxicity.

## 4. Materials and Methods

### 4.1. Cell Culture

The human Diffuse large B-cell lymphoma (DLBCL) cell lines were obtained from the Leibniz Institute DSMZ-German Collection of Microorganisms and Cell Cultures (DSMZ, Braunschweig, Germany) (Raji) and the American Type Culture Collection ATCC (Toledo), while the human leukemia cell lines (CCRF–CEM, Jurkat) were kindly provided by Elzbieta Wojdat, Institute of Immunology and Experimental Therapy, the Polish Academy of Sciences, Poland. Cells were grown in RPMI 1640 medium (4.5 g/L D-glucose) supplemented with glutamine, 10% fetal bovine serum (FBS), penicillin, and streptomycin in a humidified incubator with 5% CO_2_ at 37 °C. The culture medium was renewed every three days. Cell culture media, FBS, and antibiotics were purchased from Gibco (Thermo Fisher Scientific Inc., Waltham, MA, USA).

### 4.2. Cell Viability

To measure the metabolic activity and IC_50_ values, we used a colorimetric assay based on viable cells’ ability to convert the yellow 3-(4,5-dimethylthiazol-2-yl)-2,5-diphenyl-tetrazolium bromide (MTT) dye to purple formazan crystals. Cells were cultured at 1 × 10^4^ in 48-well plates and treated with MTX or Glu–MTX at a dose of 1µM for 48 h. Following incubation, MTT solution was added to the wells at a final concentration of 1 mg/mL for 4 h. Subsequently, the plate was centrifuged to attach the cells, the supernatant was discarded and 100 µL of DMSO was added to the cell pellet/each well. The absorbance was measured at 570 nm using the Bio-TekBioTek ELX800 multiwell reader (BioTek, Winooski, VT, USA). The experiment was conducted in triplicate and the results were expressed as the mean ± S.D. The IC50 values were calculated using CalcuSyn software (version 2.0, Biosoft, Cambridge, UK) based on concentrations 0.05–5 µM and 48 h incubation time.

### 4.3. Annexin V–FITC Assay–Flow Cytometry

The percentage of alive, early, late apoptotic, and dead cells was measured by flow cytometry. Fluorochrome-labeled Annexin V was used to identify apoptotic cells. To distinguish the necrotic and early from late apoptotic cells we used propidium iodide (PI). Early apoptotic cells exclude PI, while late apoptotic cells and necrotic cells stain positively due to the passage of PI into the nucleus. For the experiment, cells were cultured at 5 × 10^5^ in 12-well culture plates and treated with a dose of 0.1 µM MTX or Glu–MTX for 48 h. Control cells were cultured with DMSO at a final concentration of 100 nM. After 48 h, the cells were collected, centrifuged, and washed with PBS. The cell pellet of each sample was resuspended in 500 µL of 1X Binding Buffer. Then, 100 µL of each sample was mixed with 5 μL of APC fluorochrome-conjugated Annexin V and 5 μL of propidium iodide staining solution, accordingly to the staining protocol (ThermoFisher Scientific Inc., Waltham, MA, USA). After 20 min of incubation in darkness, the samples were vortexed and analyzed by a BD Accuri C6 flow cytometer (BD Biosciences, San Jose, CA, USA). The obtained results were evaluated using BD Accuri C6 plus software (version 1.0.23.1, BD Biosciences, San Jose, CA, USA).

### 4.4. Cell Cycle Analysis—Flow Cytometry

To investigate in which phase of the cell cycle, MTX and Glu–MTX arrest hematological lymphoma CCRF–CEM and Jurkat cells, cell cycle analysis with propidium iodide was performed. Propidium iodide was used as a fluorescent dye that binds to DNA. When excited by 488 nm laser light, DNA content in cell cycle analysis can be detected within the PE channel with a bandpass filter 610/10. Cells were cultured in 12-well plates in the count of 5 × 10^5^ cells per well and incubated with media containing MTX or Glu–MTX at a dose of 0.1 µM for 24 h. After incubation, the cells were collected and washed with PBS solution. After centrifugation, the cell pellet was resuspended in 500 µL of cold PBS supplemented with 2.5 µL of PerFix-nc Buffer to fix the cells (BD Biosciences, San Jose, CA, USA). After 15 min, the cells were centrifuged again. The pellet was resuspended in a solution of 0.1% (*v*/*v*) Tritox X-100, 10 mL PBS and 0.4 mL 500 µg/mL propidium iodide (PI) and incubated in the dark in protection from light. The excitation wavelength was 488 nm, and the emitted wavelength was 630 nm. Flow cytometry was performed using BD Accuri™ C6 and analyzed using dedicated software (BD Biosciences, San Jose, CA, USA).

### 4.5. Cellular Uptake—Mass Spectrometry

To measure the cellular uptake of free MTX and the MTX originated from the conjugate, liquid chromatography/electrospray ionization tandem mass spectroscopy was used. CCRF–CEM cells were seeded in density 10^6^ cells/well on a 6-well plate. Then, Glu–MTX and methotrexate were added in dose 0.1 µM for 6 h of incubation. To measure differences in cellular uptake, the cells were collected to the Eppendorf tube, centrifuged, and the medium was stored immediately at −80 °C. Next, the cell pellet was washed with 500 µL ice-cold methanol: H_2_O (3:1). The collected supernatant was again centrifuged and stored at −80 °C until the analysis.

### 4.6. Conditions of LC–ESI–MS Analysis

The Waters LC–MS system comprised an Acquity UPLC and a Xevo-G2 mass spectrometer (Milford, MA, USA). Sample compounds separation was performed on an Acquity UPLC BEH Shield column (2.1 × 50 mm, 1.7 µm). The column temperature and the autosampler temperature were kept at 40 °C and 6 °C, respectively. The mobile phase consisted of 0.1% formic acid (FA) in water was used as a mobile phase A and 0.1% FA in methanol as mobile phase B. Sample injection volume was 2 µL and the total run time of a gradient method was 6.5 min. The chromatographic method was as follows: 0.5 min—5% B, 2.0 min—40% B, 3.0 min—90% B, 4.5 min—90% B, 4.51 min—5% B. Mass spectral ionization and acquisition parameters were optimized on the Q-TOF mass spectrometer equipped with an ESI ion source in the positive ion mode. Nitrogen was used as the nebulizing and drying gas. The ion source temperature and the desolvation temperature were maintained at 120 and 400 °C. The desolvation gas flow was set at 850 L/h and the cone gas flow was 50 L/h. The capillary voltage was set at 0.5 kV. The MassLynx software (version 4.0, Waters, Milford, MA, USA) was used for data acquisition and processing.

### 4.7. Statistical Analysis

The results were presented as means from experiments performed in triplicate ± standard deviation (SD), and the statistical analysis of differences between control and treated sample was performed using an independent samples *t*-test or ANOVA Kruskal–Wallis test in the PQStat Software program (PQStat Software, Poland) The differences between groups were considered significant at *p* < 0.05.

## 5. Conclusions

In order to fulfill the hematologic malignances targeted therapies niche, we have examined the potential of glucose–methotrexate conjugate by targeting transporters that are upregulated in malignant cells. The conjugation of an antifolate metabolite with glucose seems to be a promising approach for the treatment of leukemia and lymphoma. Nevertheless, additional studies are needed to determine the therapeutic options and to overcome the limitations of the proposed therapy.

## 6. Patents

The authors are inventors on submitted patent applications (serial number P.426731).

## Data Availability

Data are contained within the article.

## Sample Availability

Samples of the compounds are not available from the authors.
